# (La_0.97_RE_0.01_Yb_0.02_)_2_O_2_S Nanophosphors Converted from Layered Hydroxyl Sulfate and Investigation of Upconversion Photoluminescence (RE=Ho, Er)

**DOI:** 10.1186/s11671-017-2277-4

**Published:** 2017-08-24

**Authors:** Ji-Guang Li, Xuejiao Wang, Weigang Liu, Qi Zhu, Xiaodong Li, Xudong Sun

**Affiliations:** 1 Key Laboratory for Anisotropy and Texture of Materials (Ministry of Education), Northeastern University, Shenyang, Liaoning 110819 China; 2 Institute of Ceramics and Powder Metallurgy, School of Materials Science and Engineering, Northeastern University, Shenyang, Liaoning 110819 China; 3 Research Center for Functional Materials, National Institute for Materials Science, Tsukuba, Ibaraki 305-0044 Japan; 4 College of New Energy, Bohai University, Jinzhou, Liaoning 121000 China; 5 School of Environmental and Chemical Engineering, Dalian University, Dalian, Liaoning 116622 China

**Keywords:** Upconversion photoluminescence, Oxysulfide, Layered hydroxyl sulfate

## Abstract

Phase-pure (La_0.97_RE_0.01_Yb_0.02_)_2_O_2_S upconversion (UC) nanophosphors (average crystallite size ~ 45 nm; RE=Ho, Er) were annealed from their hydrothermally crystallized layered hydroxyl sulfate precursors in flowing hydrogen at 1200 °C for 1 h, with water vapor as the only exhaust. Under 978-nm laser excitation (up to 2.0 W), the Ho^3+^-doped phosphor exhibited green (medium), red (weak), and near-infrared (strong) emissions at ~ 546 (^5^F_4_ → ^5^I_8_), 658 (^5^F_7_ → ^5^I_8_), and 763 nm (^5^F_4_ → ^5^I_7_), respectively, and has the stable chromaticity coordinates of about (0.30, 0.66) in the visible-light region (400–700 nm). The Er^3+^-doped UC phosphor, on the other hand, showed weak green (~ 527/549 nm, ^2^H_11/2_,^4^S_3/2_ → ^4^I_15/2_), weak red (~668/672 nm, ^4^F_9/2_ → ^4^I_15/2_), and strong near-infrared (~ 807/58 nm, ^4^I_9/2_ → ^4^I_15/2_) luminescence, whose emission color in the visible region drifted from yellowish-green [(0.36, 0.61)] to green [(0.32, 0.64)] with increasing excitation power. Analysis of the power-dependent UC luminescence found three- and two-photon processes for RE=Ho and Er, respectively, and the possible UC mechanisms were proposed.

**Electronic supplementary material:**

The online version of this article (doi:10.1186/s11671-017-2277-4) contains supplementary material, which is available to authorized users.

## Background

Upconversion (UC) phosphor is drawing considerable attention due to its unique ability to convert longer wavelength radiation into shorter wavelength fluorescence [1, 2] and is finding wide applications in the fields of solid-state lasers [3], multi-color displays [4], drug delivery [5], fluorescent biological labels [6], wavelength converters for solar cells [7], and so forth. A UC phosphor is commonly formed by doping a host lattice with a sensitizer/activator pair, where the sensitizer is usually Yb^3+^ and the activator is frequently being Ho^3+^, Er^3+^, or Tm^3+^. This is because Yb^3+^ can efficiently absorb 980-nm near-infrared laser excitation and the three types of activators have the ladder-like energy levels that are beneficial to sequential photon absorption and energy transfer [8]. The fundamentals of UC luminescence and the energy transfer in lanthanide upconversion can be found in the review articles by Auzel [9] and Dong et al. [10], respectively. Gai et al. [8] recently compiled the recent progress achieved in rare-earth micro/nanocrystals for downconversion (DC) and UC purposes, including soft chemical synthesis, luminescent properties, and biomedical applications. Wang et al. [11], on the other hand, extensively summarized in their review article the application of rare-earth ion-doped UC and DC phosphors in optical thermometry. The property of a UC phosphor is significantly affected by the type of host lattice, sensitizer/activator combination, dopant concentration, particle/crystallite morphology, crystallinity, excitation power, and the actual lattice site where the dopant ion resides [8–13]. For example, two non-equivalent Gd-activated crystallographic sites were identified in an Er^3+^-doped hexagonal Na_1.5_Gd_1.5_F_6_ phosphor, and through time-resolved spectroscopy, it was proved that the two green emissions from the ^4^S_3/2_ level of Er^3+^ separately originate from the Gd1 (540 nm) and Na2/Gd2 (550–555 nm) crystallographic sites while the 657-nm red emission from the ^4^F_9/2_ level only originates from the Na2/Gd2 site [12]. A recent study of novel Er^3+^-doped transparent Sr_0.69_La_0.31_F_2.31_ glass ceramics, on the other hand, illustrated that the spectrum split, thermal quenching ratio, population stability, and temperature sensitivity from the three thermally coupled energy levels (TCL) of ^2^H_11/2_/^4^S_3/2_, ^4^F_9/2(1)_/^4^F_9/2(2)_, and ^4^I_9/2(1)_/^4^I_9/2(2)_ are dependent on the pump power of 980 nm laser, and a new fitting method was developed to establish the relation between fluorescence intensity ratios and temperature [13]. Rare-earth (RE) halides (such as NaYF_4_:Yb/Er) are currently the most efficient UC phosphors owing to their low phonon energies (ℏω < 400 cm^−1^) [8, 10, 11, 14], though the toxic raw materials involved in the synthesis and the air sensitivity of many halides restrain their application and production. Another type of widely investigated UC phosphors is RE_2_O_3_ (such as Y_2_O_3_:Yb/Er), whose relatively high phonon energy (ℏω ~ 600 cm^−1^; ~ 591 cm^−1^ for Y_2_O_3_ and 612 cm^−1^ for Lu_2_O_3_) [15], however, lowers the efficiency of UC luminescence due to photon-phonon coupling. From the view point of biocompatibility, Li et al. [16] synthesized Yb^3+^- and Ho^3+^-codoped fluorapatite crystals (nanorods of 16 by 286 nm) via hydrothermal reaction, and UC luminescence of Ho^3+^ at 543 and 654 nm was attained through a two-photon process under 980-nm laser excitation. The crystals also exhibited clear fluorescent cell imaging after the surfaces were grafted with hydrophilic dextran [16].

RE_2_O_2_S oxysulfide is an important family of compounds in the phosphor field and can be advantageous over oxide for luminescent applications. For example, the occurrence of S^2−^ → Eu^3+^ charge transfer transition in Eu^3+^-activated RE_2_O_2_S significantly extends the effective excitation wavelength to ~ 400 nm [17–19], which makes the phosphor useful as the red component in near-UV (365–410 nm)-excited white LEDs as reviewed by Ye et al. [20]. The most matured technique to synthesize RE_2_O_2_S is solid-state reaction, which has the advantages of high yield and convenience, but high reaction temperature, uncontrollable product morphology, and especially the employment of environmentally harmful sulfur sources are apparent shortcomings [21–23]. Sulfurization of RE_2_O_3_ by H_2_S or CS_2_ gas at an elevated temperature [24–26] is another frequently used strategy to produce RE_2_O_2_S. Since the methodology for controlled synthesis of RE_2_O_3_ is rich and well developed, RE_2_O_2_S with various particle morphologies has thus been produced through the sulfurization route, though the complicated procedures are less feasible for industrial production. Other techniques for RE_2_O_2_S synthesis may include precipitation [27], hydrothermal reaction [28], two-step solution gel polymer thermolysis [29], gelatin-templated synthesis [30], gel thermolysis [31], solvothermal pressure-relief synthesis [32], and combustion [33]. The involvement of harmful sulfur sources or by-products (such as C_2_S, H_2_S, and thiourea) is, however, still hard to avoid. The appearance of sulfate-type layered rare-earth hydroxide (RE_2_(OH)_4_SO_4_∙2H_2_O, SO_4_^2−^-LREH) in 2010 [34] provided a unique chance to solve the aforementioned issues, since this group of compounds has exactly the same RE/S molar ratio of RE_2_O_2_S. Homogenous hydrolysis of RE_2_(SO_4_)_3_·8H_2_O in the presence of Na_2_SO_4_ and hexamethylenetetramine (C_6_H_12_N_4_) is the classic technique to produce SO_4_^2−^-LREH but is limited to RE=Pr–Tb in the lanthanide family [34]. We extended the group of compounds to RE=La–Dy via reacting aqueous solutions of RE(NO_3_)_3_·*n*H_2_O and (NH_4_)_2_SO_4_ under hydrothermal conditions [17–19] and subsequently manifested that RE_2_O_2_S can be facilely produced through thermolysis of SO_4_^2−^-LREH in a reducing atmosphere [17–19]. RE_2_O_2_S was recently identified to have relatively low phonon energy (ℏω ~ 500 cm^−1^) [1], good chemical stability, and particularly high UC efficiency comparable to halides [35, 36], but the study on this type of promising UC phosphors is yet far from sufficiency [8, 10, 11, 37, 38]. La^3+^ does not have unoccupied 4*f* sub-orbital and is optically inert, and thus, its compounds are suitable host lattices for luminescence. We thus synthesized in this work La_2_O_2_S:Yb/RE UC phosphors (RE=Ho, Er) via annealing the hydrothermally crystallized SO_4_^2−^-LREH in flowing H_2_, and the luminescent properties and UC processes were elaborated in detail.

## Methods

The starting materials of RE(NO_3_)_3_·6H_2_O (RE=La, Ho, Er, and Yb; > 99.99% pure), (NH_4_)_2_SO_4_ (> 99.5% pure), and NH_3_·H_2_O solution (28%, ultrahigh purity) were purchased from Kanto Chemical Co., Inc. (Tokyo, Japan), and were used as received. Yb^3+^/Ho^3+^- and Yb^3+^/Er^3+^-doped La_2_(OH)_4_SO_4_·2H_2_O was separately synthesized via hydrothermal reaction. The dopant contents are 2 at.% for Yb^3+^ and 1 at.% for both Ho^3+^ and Er^3+^ according to the literature [39]. In a typical synthesis [17], 6 mmol of (NH_4_)_2_SO_4_ was dissolved in 60 ml of an aqueous solution of the rare earths (0.1 mol/L for total RE^3+^), followed by dropwise addition of NH_3_·H_2_O until pH = 9. After continuous stirring for 15 min, the resultant suspension was transferred into a Teflon-lined autoclave of 100-ml capacity for 24 h of hydrothermal crystallization in an electric oven preheated to 100 °C. The resultant product was collected via centrifugation, washed with filtered water three times and ethanol once, and finally dried in air at 70 °C for 24 h. The La_2_O_2_S:Yb/RE UC phosphors were then annealed from their SO_4_^2−^-LREH precursors in flowing H_2_ (200 mL/min) at 1200 °C for 1 h, with a heating rate of 5 °C/min in the ramp stage.

Phase identification was performed via X-ray diffractometry (XRD; Model RINT2200, Rigaku, Tokyo, Japan) under 40 kV/40 mA, using nickel-filtered Cu-*K*α radiation (λ = 0.15406 nm) and a scanning speed of 1°/min. Structure parameters of the products were derived from the XRD data using the TOPAS software [40]. Particle morphology was observed by field emission scanning electron microscopy (FE-SEM; Model S-5000, Hitachi, Tokyo) under an acceleration voltage of 10 kV. UC luminescence spectra were obtained at room temperature using an FP-6500 fluorospectrophotometer (JASCO, Tokyo) under 978-nm near-infrared laser excitation of the phosphors with a continuous wavelength (CW) laser diode (Model KS3–12322-105, BWT Beijing Ltd., Beijing, China). The signal/noise ratio (S/N) of the spectrometer is of ≥ 200, and the sensitivity was set low due to the strong UC luminescence of the phosphors. The experimental setup can be found in Additional file 1: Figure S1.

## Results and Discussion

**Fig. 1:**
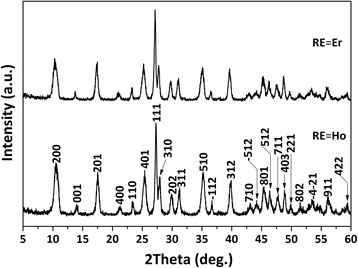
XRD patterns of the (La_0.97_RE_0.01_Yb_0.02_)_2_(OH)_4_SO_4_·2H_2_O layered compounds obtained via hydrothermal reaction at 100 °C and pH = 9 for 24 h

Figure 1 shows XRD patterns of the hydrothermal products, where it is seen that in each case, all the diffraction peaks can be well indexed with the layered compound of La_2_(OH)_4_SO_4_·2H_2_O [17, 18]. In an aqueous solution containing SO_4_^2−^, the rare-earth (RE) cations would undergo hydration and partial hydrolysis to form the complex ions of [RE(OH)_*x*_(H_2_O)_*y*_(SO_4_)_*z*_]^3-*x*-2*z*^ [17–19]. Either a higher temperature or solution pH will promote RE^3+^ hydrolysis, leading to more OH^−^ while less SO_4_^2−^ (smaller SO_4_^2−^/OH^−^ molar ratio) in the complex ion. Under the optimized hydrothermal conditions of 100 °C and pH = 9 [17–19], the complex ion may have a proper SO_4_^2−^/OH^−^ molar ratio, and thus, the aimed SO_4_^2−^-LREH compound can be crystallized via condensation reactions. The structure parameters of the hydrothermal products are summarized in Table 1. It is clear that (La_0.97_Ho_0.01_Yb_0.02_)_2_(OH)_4_SO_4_·2H_2_O has larger lattice constants (*a*, *b*, *c*) and cell volume (*V*) than (La_0.97_Er_0.01_Yb_0.02_)_2_(OH)_4_SO_4_·2H_2_O. This is understandable in view that Ho^3+^ (1.072 Å for CN = 9) is larger than Er^3+^ (1.062 Å for CN = 9). Both the products have smaller cell constants and cell volume than the un-doped La_2_(OH)_4_SO_4_·2H_2_O (SO_4_^2−^-LLaH), in accordance with the fact that La^3+^ is the largest (1.216 Å for CN = 9) among the four types of RE ions. The differing cell parameters provided direct evidence of solid-solution formation.

**Table 1:** Structure parameters of the (La_0.97_RE_0.01_Yb_0.02_)_2_(OH)_4_SO_4_·2H_2_O layered compounds obtained in this work and La_2_(OH)_4_SO_4_∙2H_2_O·(SO_4_^2−^-LLaH) [17]

Sample	Sp.Gr.	*a*, Å	*b*, Å	*c*, Å	*β*, °	*V*, Å^3^
RE=Ho	*C*2*/m*	16.8447	3.9268	6.4227	90.535	424.816
RE=Er	*C*2*/m*	16.8411	3.9256	6.4217	90.522	424.530
SO_4_^2−^-LLaH	*C*2*/m*	16.8847	3.9420	6.4359	90.454	428.363

**Fig. 2:**
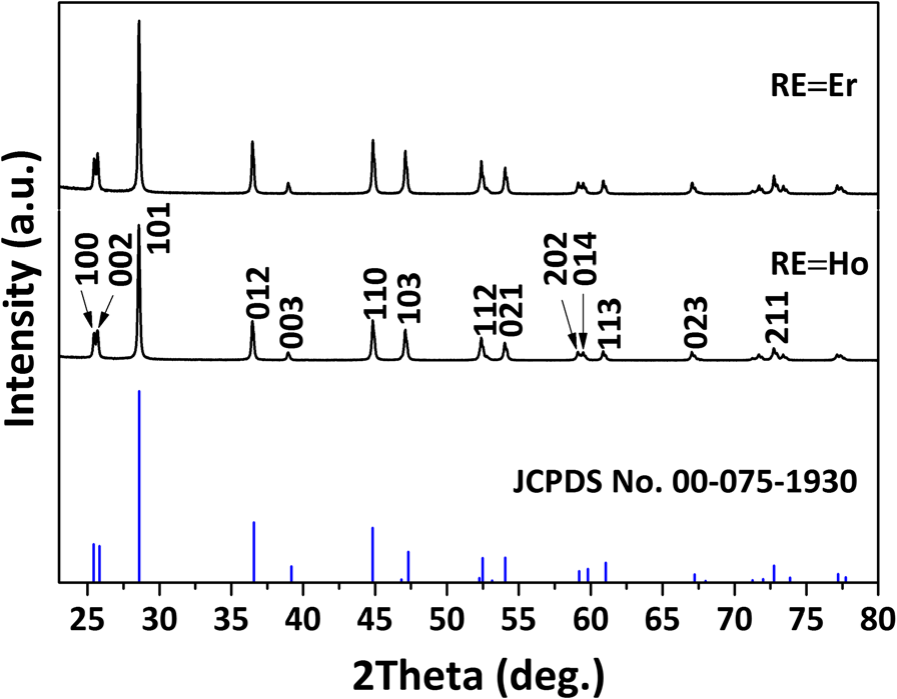
XRD patterns of the (La_0.97_RE_0.01_Yb_0.02_)_2_O_2_S upconversion phosphors calcined from their layered precursors in flowing H_2_ (200 ml/min) at 1200 °C for 1 h. The standard diffractions of La_2_O_2_S are included as bars for comparison

Figure 2 shows XRD patterns of the products annealed from their SO_4_^2−^-LREH precursors at 1200 °C for 1 h in flowing H_2_. The diffraction peaks can be fully indexed with the hexagonal structured La_2_O_2_S in each case (space group: *P-*3*m*1; JCPDS card no. 00-075-1930). SO_4_^2−^-LLaH would decompose to La_2_O_2_SO_4_ up to 1200 °C in air through the reactions of La_2_(OH)_4_SO_4_·2H_2_O → La_2_(OH)_4_SO_4_ + 2H_2_O (dehydration) and La_2_(OH)_4_SO_4_ → La_2_O_2_SO_4_ + 2H_2_O (dehydroxylation) [17]. In a H_2_ atmosphere, the S^6+^ in SO_4_^2−^ would be reduced to S^2−^ following the reaction of La_2_O_2_SO_4_ + 4H_2_ → La_2_O_2_S + 4H_2_O, and thus, La_2_O_2_S can be resulted with water vapor as the only by-product [17]. The lattice parameters and cell volume of (La_0.97_RE_0.01_Yb_0.02_)_2_O_2_S are shown in Table 2 together with those of La_2_O_2_S [17]. The decreasing cell dimension towards a smaller RE^3+^ indicates the successful formation of solid solution.

**Table 2:** Structure parameters of the (La_0.97_RE_0.01_Yb_0.02_)_2_O_2_S phosphors obtained in this work and those of La_2_O_2_S [17]

Sample	Sp.Gr.	*a*, Å	*b*, Å	*c*, Å	*V*, Å^3^
RE=Ho	*P-*3*m*1	4.0413	4.0413	6.9355	98.096
RE=Er	*P-*3*m*1	4.0410	4.0410	6.9354	98.080
La_2_O_2_S	*P-*3*m*1	4.0520	4.0520	6.9463	98.770

**Fig. 3:**
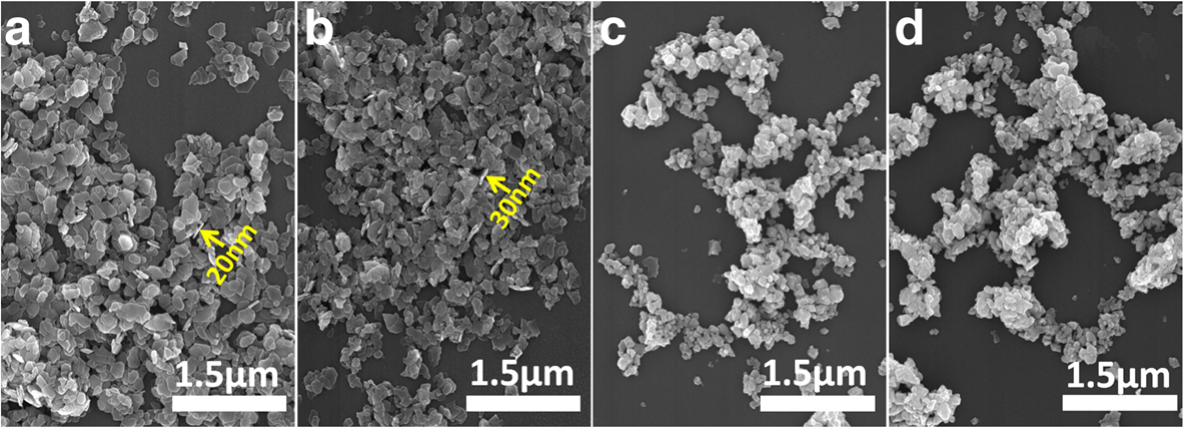
FE-SEM particle morphologies of the (La_0.97_Ho_0.01_Yb_0.02_)_2_(OH)_4_SO_4_·2H_2_O (**a**) and (La_0.97_Er_0.01_Yb_0.02_)_2_(OH)_4_SO_4_·2H_2_O (**b**) layered precursors and the (La_0.97_Ho_0.01_Yb_0.02_)_2_O_2_S (**c**) and (La_0.97_Er_0.01_Yb_0.02_)_2_O_2_S (**d**) upconversion phosphors

Figure 3 shows the particle morphology of the layered precursors and the resultant UC phosphors. It is seen that the SO_4_^2−^-LREH crystallized as nanoplates of ~ 150–550 nm in lateral size and ~ 20–30 nm in thickness. The nanoplates underwent significant disintegration upon calcination at 1200 °C to yield rounded particles. The average crystallite size was assayed with the Scherrer equation to be ~ 45 nm for the UC phosphors.

**Fig. 4:**
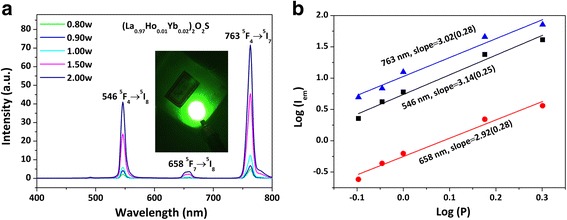
Upconversion luminescence spectra (**a**) and the relationship between log(*I*_em_) and log(*P*) (**b**) for the (La_0.97_Ho_0.01_Yb_0.02_)_2_O_2_S phosphor, where *I*_em_ and *P* are the emission intensity and excitation power (in watt), respectively. The inset in **a** is a photograph showing the appearance of strong UC emission under 50 mW of 978-nm laser excitation

Figure 4a shows UC luminescence spectra of the (La_0.97_Ho_0.01_Yb_0.02_)_2_O_2_S phosphor under 978-nm laser excitation. The emissions at ~ 546, 658, and 763 nm are attributed to the ^5^F_4_ → ^5^I_8_, ^5^F_7_ → ^5^I_8_, and ^5^F_4_ → ^5^I_7_ transitions of Ho^3+^, respectively [36], with the 763-nm NIR emission being predominant. The sensitizing effect of Yb^3+^ is significant, and ~ 15 and 20 times stronger green (546 nm) and NIR (763 nm, not sensitive to human eyes) emissions, respectively, were produced by codoping of 2 at.% Yb^3+^ (Additional file 1: Figure S2a). Under 50-mW laser pumping, vivid and strong green emission was observed for the (La_0.97_Ho_0.01_Yb_0.02_)_2_O_2_S phosphor with naked eyes, as shown in the insert in Fig. 4a. In spite of the excitation power, the CIE color coordinates calculated from the emission spectra in the visible light region (400–700 nm) are stable at about (0.30, 0.66), typical of a vivid green color (Additional file 1: Table S1 and Figure S3).

In general, the number of photons required to populate the upper emitting state under unsaturated condition can be obtained from the relation *I*_em_∝*P* ^*n*^ [41], where *I* is the luminescence intensity, *P* the pumping power, and *n* the number of laser photons. Figure 4b shows the log(*I*_em_)-log(*P*) plot of the above relation, from which the *n* value was determined from the slope of the linear fitting to be ~3.02, 3.14, and 2.92 (approximately 3) for the UC emissions peaked at ~546, 658, and 763 nm, respectively. The results thus suggest that a three-photon process was involved to generate the observed UC luminescence.

**Fig. 5:**
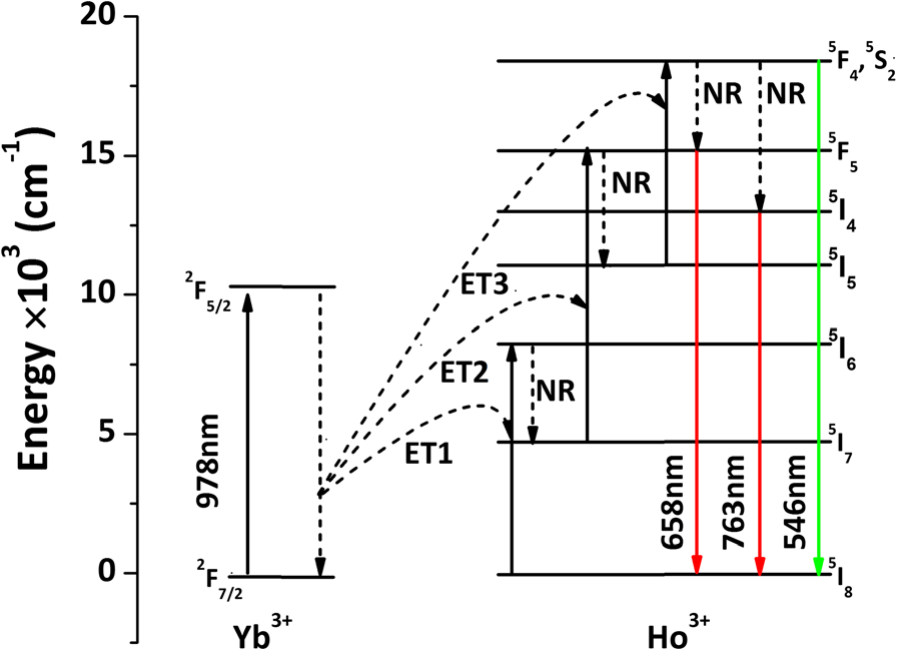
A schematic illustration of the energy levels and UC processes for the (La_0.97_Ho_0.01_Yb_0.02_)_2_O_2_S phosphor

In principle, three basic population mechanisms, namely excited state absorption (ESA), energy transfer (ET), and photon avalanche, may be involved in an UC process [8–10]. Since no power threshold was observed in the range of this study, the photon avalanche mechanism can be neglected. The energy diagram of Yb^3+^/Ho^3+^ in La_2_O_2_S has rarely been reported and is not available to us for comparison. Nonetheless, UC luminescence involving three phonons was seen from the previous work on Yb^3+^/Ho^3+^-codoped other material systems [42, 43]. Therefore, the energy diagram and UC process of (La_0.97_Ho_0.01_Yb_0.02_)_2_O_2_S were constructed in Fig. 5 by referring to these previous studies and are detailed below: (1) excitation of Yb^3+^ by laser photons [ESA; ^2^F_7/2_(Yb^3+^) + *hν* (978 nm) → ^2^F_5/2_(Yb^3+^)]; (2) population of the ^5^I_6_ energy level of Ho^3+^ after Yb^3+^ absorbing the first laser photon and transferring energy to Ho^3+^ [ET1; ^2^F_5/2_(Yb^3+^) + ^5^I_8_(Ho^3+^) → ^2^F_7/2_(Yb^3+^) + ^5^I_6_(Ho^3+^)]; (3) non-radiative (NR) relaxation to the ^5^I_7_ level of Ho^3+^ [NR; ^5^I_6_(Ho^3+^) ~ ^5^I_7_(Ho^3+^)]; (4) excitation of Ho^3+^ from the ^5^I_7_ to ^5^F_5_ level after Yb^3+^ absorbing the second laser photon and transferring energy to Ho^3+^ [ET2; ^2^F_5/2_(Yb^3+^) + ^5^I_7_(Ho^3+^) → ^2^F_7/2_(Yb^3+^) + ^5^F_5_(Ho^3+^)]; (5) NR relaxation to the ^5^I_5_ level of Ho^3+^ [^5^F_5_(Ho^3+^) ~ ^5^I_5_(Ho^3+^)]; (6) excitation of Ho^3+^ from the ^5^I_5_ to ^5^F_4_/^5^S_2_ level after Yb^3+^ absorbing the third laser photon and transferring energy to Ho^3+^ [ET3; ^2^F_5/2_(Yb^3+^) + ^5^I_5_(Ho^3+^) → ^2^F_7/2_(Yb^3+^) + ^5^F_4_/^5^S_2_(Ho^3+^)]; and (7) back-jumping of the excited electrons from the populated ^5^F_4_/^5^S_2_ level to the ^5^I_8_ ground state to produce the green emission (~546 nm; ^5^F_4_,^5^S_2_ → ^5^I_8_). The electrons can also relax to the ^5^F_5_ and ^5^I_4_ levels via NR processes, from which the red (~658 nm; ^5^F_5_ → ^5^I_8_) and near-infrared (~763 nm; ^5^I_4_ → ^5^I_8_) emissions were resulted. The strong near-infrared UC emission at ~763 nm may imply that NR relaxation to the ^5^I_8_ energy level is significant.

**Fig. 6:**
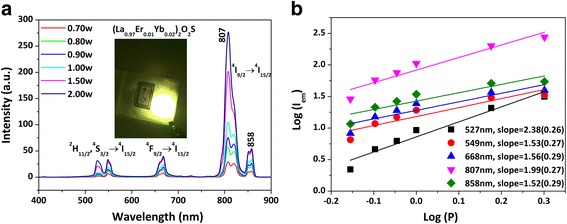
Upconversion luminescence spectra (**a**) and the relationship between log(*I*_em_) and log(*P*) (**b**) for the (La_0.97_Er_0.01_Yb_0.02_)_2_O_2_S phosphor, where *I*_em_ and *P* are the emission intensity and excitation power (in watt), respectively. The inset in **a** is a photograph showing the strong UC emission of (La_0.97_Er_0.01_Yb_0.02_)_2_O_2_S under 50 mW of 978-nm laser excitation

The ^2^F_5/2_ → ^2^F_7/2_ emission transition of Yb^3+^ and the ^4^I_15/2_ → ^4^I_11/2_ excitation transition of Er^3+^ have well-matching energies, which makes Yb^3+^/Er^3+^ the most widely investigated activator/sensitizer pair for UC luminescence in various types of host lattices [8–13]. Similar to Ho^3+^, the UC emission of Er^3+^ was also dramatically enhanced by Yb^3+^ codoping (Additional file 1: Figure S2b). Taking the 527-nm green emission for example, 2 at.% of Yb^3+^ improved the Er^3+^ luminescence by a factor of ~ 14. Under 978-nm laser excitation, the (La_0.97_Er_0.01_Yb_0.02_)_2_O_2_S UC phosphor exhibits emission bands in the green (~ 527 and 549 nm), red (~ 668 and 672 nm), and near-infrared (~ 807 and 858 nm) regions (Fig. 6a), which are arising from the ^2^H_11/2_/^4^S_3/2_ → ^4^I_15/2_, ^4^F_9/2_ → ^4^I_15/2_, and ^4^I_9/2_ → ^4^I_15/2_ transitions of Er^3+^, respectively [32]. The color coordinates determined for the UC luminescence in the visible light region (400–700 nm) drifted from the yellowish-green [(0.36, 0.61)] to green [(0.32, 0.64)] region in the CIE chromaticity diagram along with increasing excitation power from 0.7 to 2.0 W (Additional file 1: Figure S3b and Table S2). The color change also agrees well with the gradually larger intensity ratio of the green to red emissions (*I*_549_/*I*_668_ and *I*_527_/*I*_668_, Additional file 1: Table S3) under a higher excitation power. Excitation-power-dependent emission-color tuning of the Yb^3+^/Er^3+^ pair was previously observed in Y_2_O_2_S [44]. The number of pumping photons required to populate the emitting states was derived from the slope of the log(*I*_em_)-log(*P*) plot (Fig. 6b), and the three groups of emissions were found to have the similar *n* values of ~ 2. This indicates that a two-phonon process is largely responsible for the observed UC luminescence.

**Fig. 7:**
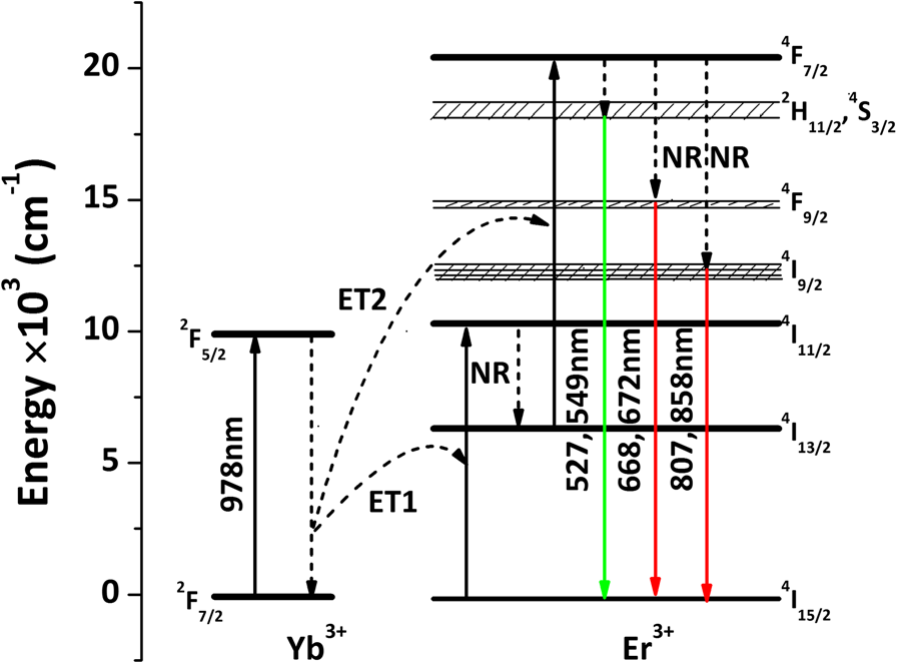
A schematic illustration of the energy levels and UC processes for (La_0.97_Er_0.01_Yb_0.02_)_2_O_2_S phosphor

The energy diagram and photon process leading to the UC luminescence of (La_0.97_Er_0.01_Yb_0.02_)_2_O_2_S are schematically shown in Fig. 7. Excited-state absorption and Yb^3+^ → Er^3+^ energy transfer excitation are mainly involved in the UC mechanism, with the latter being dominant [8–14, 39, 43]. Upon excitation with 978-nm laser, the ^2^F_7/2_ ground state electrons of Yb^3+^ are pumped to the ^2^F_5/2_ excited state (ESA). Since the ^2^F_5/2_ level of Yb^3+^ and the ^4^I_11/2_ level of Er^3+^ are matching well with each other, energy transfer from Yb^3+^ to Er^3+^ readily takes place. The Er^3+^ electrons can thus be excited from the ^4^I_15/2_ ground state to the ^4^I_11/2_ level with the energy transferred from Yb^3+^ (one photon, ET1). The absorption cross section of Er^3+^ is smaller than that of Yb^3+^ at ~ 980 nm [42, 45], so energy transfer (ET) dominates the real excitation of Er^3+^. The excitation energy at the ^4^I_11/2_ level may non-radiatively (NR) relax to the ^4^I_13/2_ level, from which the electrons can be excited to the ^4^F_7/2_ state by ET of a second laser photon (ET2). After NR processes, the three groups of emissions (Fig. 6a) can then be produced via the electronic transitions shown in Fig. 7. The photon reactions of the whole UC process can be presented as follows: (1) ^2^F_7/2_(Yb^3+^) + *hν* (978 nm) → ^2^F_5/2_(Yb^3+^) and ^4^I_15/2_(Er^3+^) + *h*ν (978 nm) → ^4^I_11/2_(Er^3+^); (2) ^2^F_5/2_(Yb^3+^) + ^4^I_15/2_(Er^3+^) → ^2^F_7/2_(Yb^3+^) + ^4^I_11/2_(Er^3+^); (3) ^4^I_11/2_(Er^3+^) ~ ^4^I_13/2_(Er^3+^); (4) ^2^F_5/2_(Yb^3+^) + ^4^I_13/2_(Er^3+^) → ^2^F_7/2_(Yb^3+^) + ^4^F_7/2_(Er^3+^); (5) ^4^F_7/2_(Er^3+^) ~ ^2^H_11/2_/^4^S_3/2_(Er^3+^), ^4^F_9/2_(Er^3+^), and ^4^I_9/2_(Er^3+^); and (6) ^2^H_11/2_/^4^S_3/2_(Er^3+^) → ^4^I_15/2_(Er^3+^) + *hν* (~527 and 549 nm), ^4^F_9/2_(Er^3+^) → ^4^I_15/2_(Er^3+^) + *hν* (~668 and 672 nm), and ^4^I_9/2_(Er^3+^) → ^4^I_15/2_(Er^3+^) + *hν* (~807 and 858 nm). The aforementioned emission color change may suggest a more efficient population of the ^4^F_7/2_(Er^3+^) energy level under a higher excitation power, and the ^4^F_7/2_(Er^3+^) ~ ^2^H_11/2_/^4^S_3/2_(Er^3+^) NR process becomes successively stronger than ^4^F_7/2_(Er^3+^) ~ ^4^F_9/2_(Er^3+^).

## Conclusions

(La_0.97_RE_0.01_Yb_0.02_)_2_O_2_S upconversion (UC) nanophosphors (RE=Ho, Er) were successfully produced via thermal decomposition of their layered hydroxyl sulfate precursors in flowing H_2_ at 1200 °C, with water vapor as the only exhaust. The precursors crystallized as nanoplates with the lateral sizes of ~ 150–550 nm and thicknesses of ~ 20–30 nm, which disintegrated into rounded nanoparticles (average crystallite size: ~ 45 nm) upon thermal decomposition. The oxysulfide phosphors exhibit strong UC luminescence under 978-nm laser excitation, through a three-photon process for Ho^3+^ and a two-photon process for Er^3+^. For the UC luminescence in the visible region (400–700 nm), the chromaticity coordinates of (La_0.97_Ho_0.01_Yb_0.02_)_2_O_2_S are stable at around (0.30, 0.66), while those of (La_0.97_Er_0.01_Yb_0.02_)_2_O_2_S changed from about (0.36, 0.61) to (0.32, 0.64) along with the excitation power increasing from 0.7 to 2 W.

## Additional files

Additional file 1: Figure S1. Configuration of laser pumping and UC measurement in JASCO FP-6500 sample chamber. **Figure S2.** UC luminescence comparison of (La,RE,Yb)_2_O_2_S and (La,RE)_2_O_2_S under the excitation of 978-nm laser. **Table S1.** CIE chromaticity coordinates of the (La_0.97_Ho_0.01_Yb_0.02_)_2_O_2_S UC phosphor under different excitation power. **Figure S3.** CIE chromaticity diagram for the UC emissions of (La_0.97_Ho_0.01_Yb_0.02_)_2_O_2_S (a) and (La_0.97_Er_0.01_Yb_0.02_)_2_O_2_S (b). **Table S2.** CIE chromaticity coordinates of the (La_0.97_Er_0.01_Yb_0.02_)_2_O_2_S UC phosphor under different excitation power. **Table S3.** Excitation power dependence of the I_549_/I_668_ and I_527_/I_668_ intensity ratios for the (La_0.97_Er_0.01_Yb_0.02_)_2_O_2_S UC phosphor. (DOC 2407 kb)
