# Supplementary material for: (La0.97RE0.01Yb0.02)2O2S Nanophosphors Converted from Layered Hydroxyl Sulfate and Investigation of Upconversion Photoluminescence (RE=Ho, Er)
Source: Nanoscale Res Lett. 2017 Aug 24;12:508. doi: 10.1186/s11671-017-2277-4 (PMC5570760; doi:10.1186/s11671-017-2277-4)
Supplement: Supplementary file 1 — Configuration of laser pumping and UC measurement in JASCO FP-6500 sample chamber. Figure S2. UC luminescence comparison of (La,RE,Yb)2O2S and (La,RE)2O2S under the excitation of 978-nm laser. Table S1. CIE chromaticity coordinates of the (La0.97Ho0.01Yb0.02)2O2S UC phosphor under different excitation power. Figure S3. CIE chromaticity diagram for the UC emissions of (La0.97Ho0.01Yb0.02)2O2S (a) and (La0.97Er0.01Yb0.02)2O2S (b). Table S2. CIE chromaticity coordinates of the (La0.97Er0.01Yb0.02)2O2S UC phosphor under different excitation power. Table S3. Excitation power dependence of the I549/I668 and I527/I668 intensity ratios for the (La0.97Er0.01Yb0.02)2O2S UC phosphor. (DOC 2407 kb) [file 11671_2017_2277_MOESM1_ESM.doc]

**Supporting Information**

**(La_0.97_RE_0.01_Yb_0.02_)_2_O_2_S nanophosphors converted from layered hydroxyl sulfate and investigation of upconversion photoluminescence (RE＝Ho, Er)**

Ji-Guang Li,^a,b,c*^ Xuejiao Wang,^c,d*^ Weigang Liu,^a,b^ Qi Zhu,^a,b^ Xiaodong Li,^a,b^ and Xudong Sun^a,b,e^

^a^Key Laboratory for Anisotropy and Texture of Materials (Ministry of Education), Northeastern University, Shenyang, Liaoning 110819, China

^b^Institute of Ceramics and Powder Metallurgy, School of Materials Science and Engineering, Northeastern University, Shenyang, Liaoning 110819, China

^c^Research Center for Functional Materials, National Institute for Materials Science, Tsukuba, Ibaraki 305-0044, Japan

^d^College of New Energy, Bohai University, Jinzhou, Liaoning 121000, China

^e^School of Environmental and Chemical Engineering, Dalian University, Dalian, Liaoning 116622, China

^*^Corresponding author

National Institute for Materials Science

Tel: +81-29-860-4394

E-mail: [LI.Jiguang@nims.go.jp](mailto:LI.Jiguang@nims.go.jp)


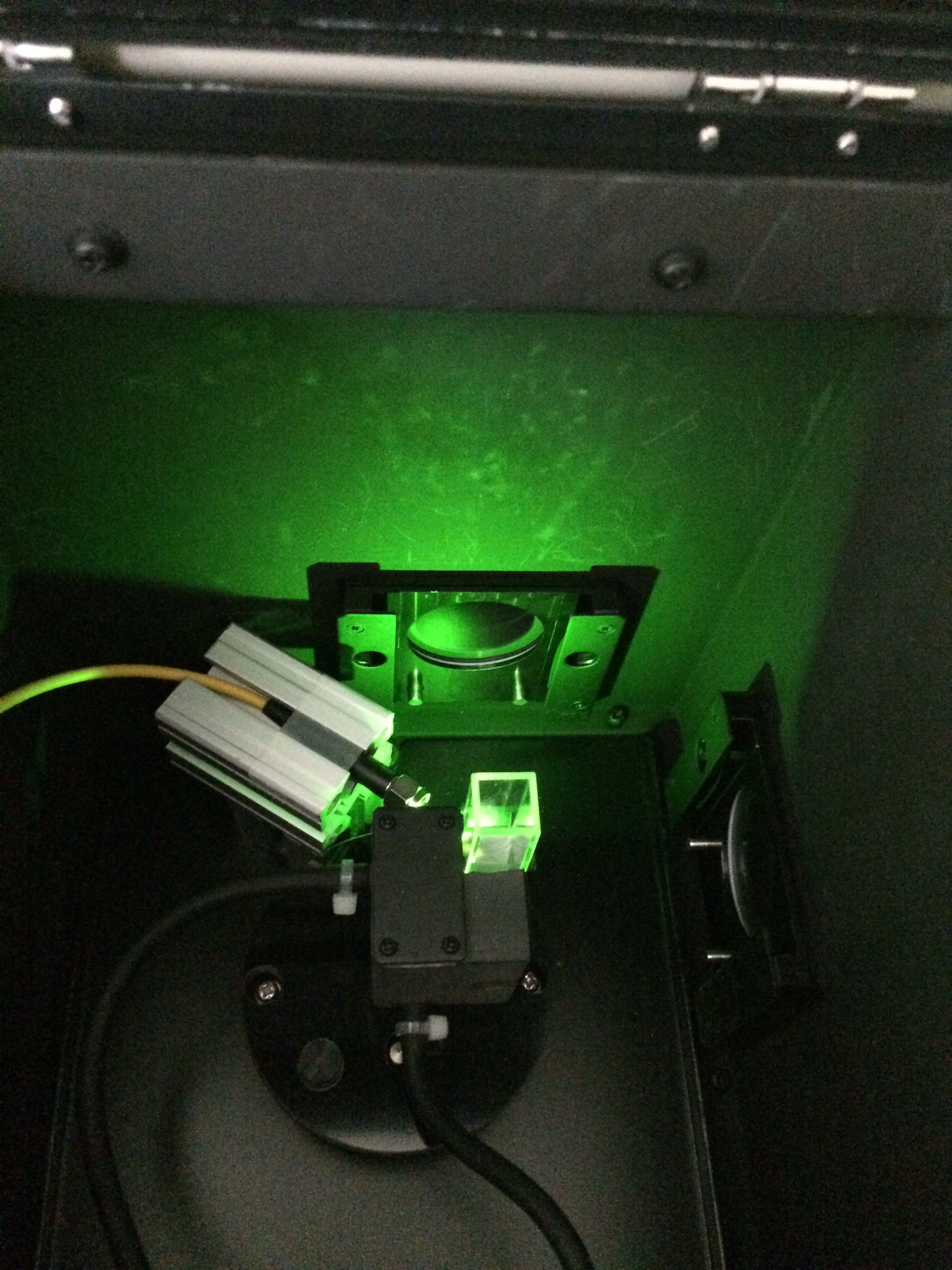


**Figure S1** Configuration of laser pumping and UC measurement in JASCO FP-6500 sample chamber.

**
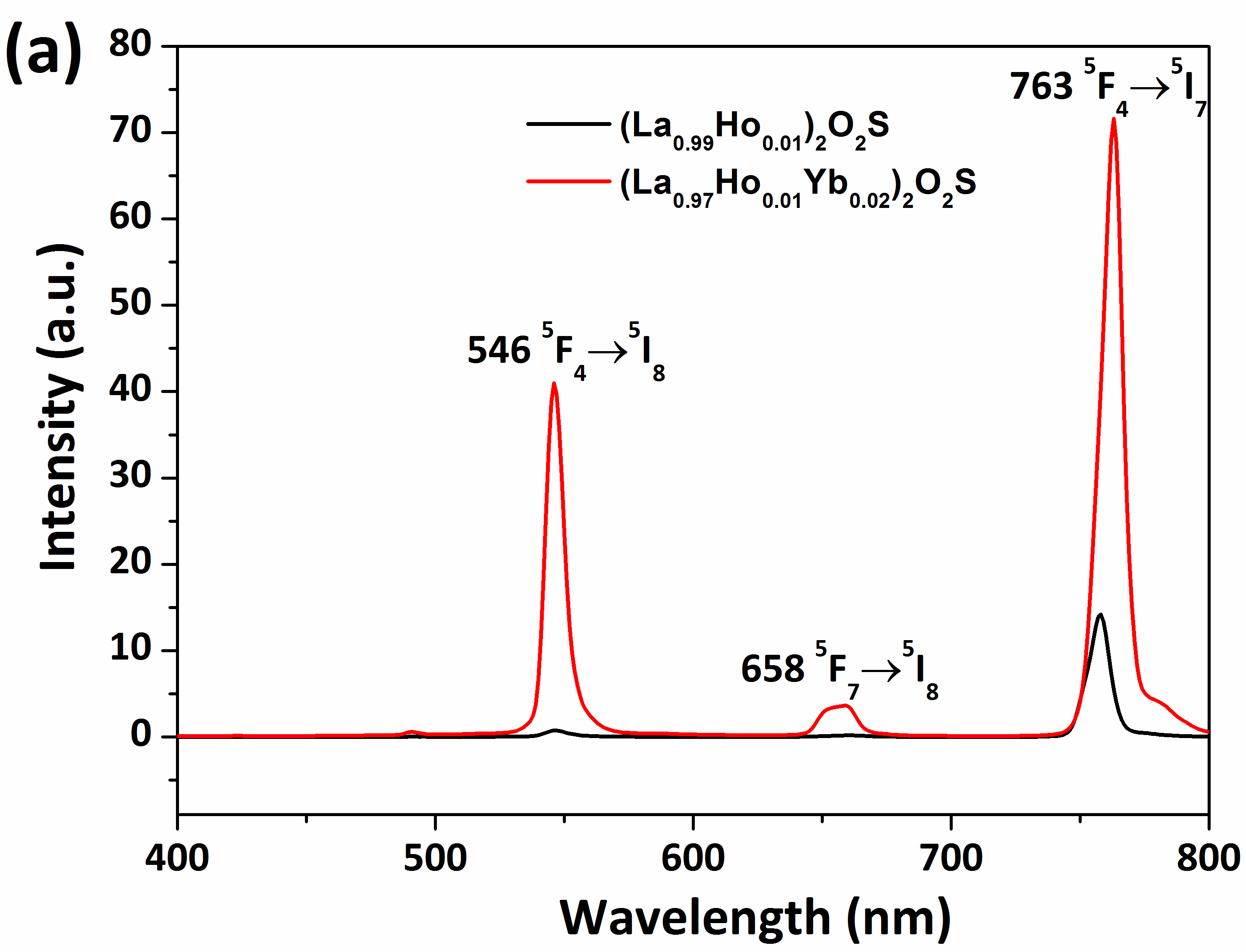
**

**
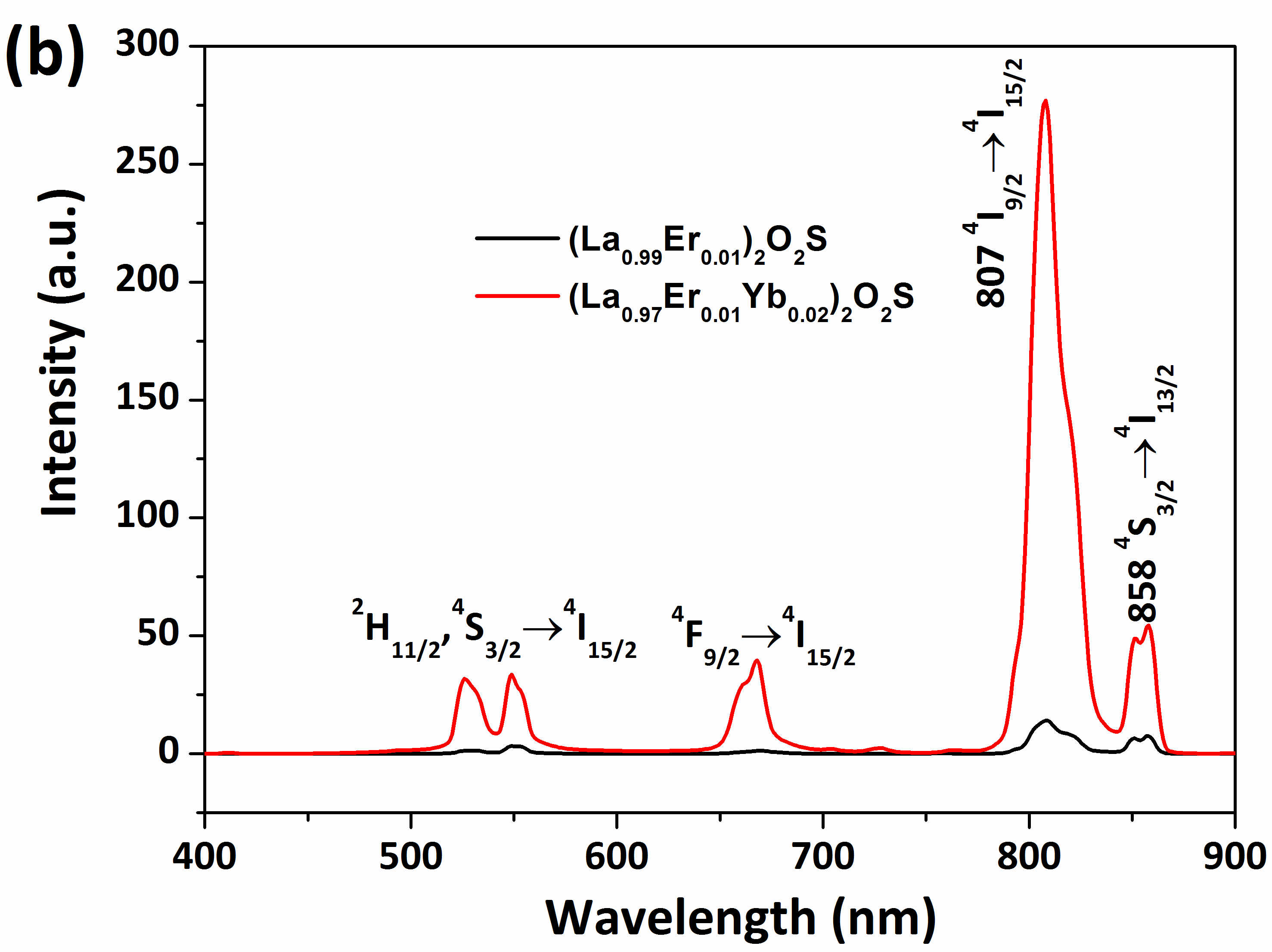
**

**Figure S2** UC luminescence comparison of (La,RE,Yb)_2_O_2_S and (La,RE)_2_O_2_S under the excitation of 978 nm laser.

**
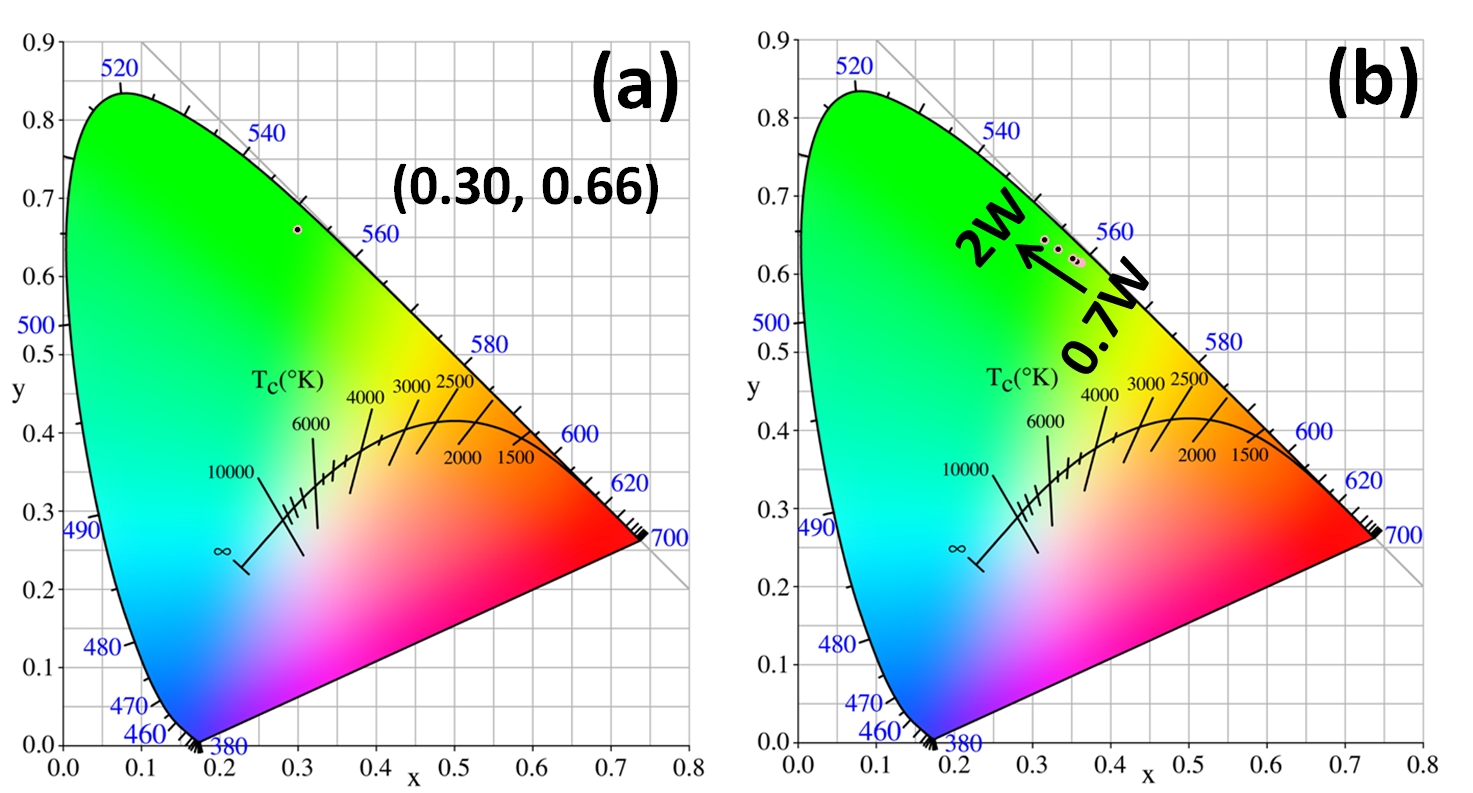
**

**Figure S3** CIE chromaticity diagram for the UC emissions of (La_0.97_Ho_0.01_Yb_0.02_)_2_O_2_S (a) and (La_0.97_Er_0.01_Yb_0.02_)_2_O_2_S (b).

**Table S1** CIE chromaticity coordinates of the (La_0.97_Ho_0.01_Yb_0.02_)_2_O_2_S UC phosphor under different excitation power.

| Excitation power (W) | CIE chromaticity |
| --- | --- |
| 0.8 | (0.3053, 0.6677) |
| 0.9 | (0.3064, 0.6678) |
| 1 | (0.3078, 0.6692) |
| 1.5 | (0.3052, 0.6647) |
| 2 | (0.3045, 0.6643) |

**Table S2** CIE chromaticity coordinates of the (La_0.97_Er_0.01_Yb_0.02_)_2_O_2_S UC phosphor under different excitation power.

| Excitation power (W) | CIE chromaticity |
| --- | --- |
| 0.7 | (0.3626, 0.6144) |
| 0.8 | (0.3598, 0.6153) |
| 0.9 | (0.3573, 0.6164) |
| 1 | (0.3516, 0.6194) |
| 1.5 | (0.3331, 0.6320) |
| 2 | (0.3154, 0.6439) |

**Table S3** Excitation power dependence of the I_549_/I_668_ and I_527_/I_668_ intensity ratios for the (La_0.97_Er_0.01_Yb_0.02_)_2_O_2_S UC phosphor

| Excitation power (W) | I_549_/I_668_ | I_527_/I_668_ |
| --- | --- | --- |
| 0.7 | 0.793723 | 0.268266 |
| 0.8 | 0.782221 | 0.307791 |
| 0.9 | 0.783520 | 0.328279 |
| 1 | 0.785718 | 0.378382 |
| 1.5 | 0.815748 | 0.565825 |
| 2 | 0.846931 | 0.791176 |
